# An integrative review of how midwives are screening and assessing for trauma in women within perinatal services

**DOI:** 10.1371/journal.pone.0327253

**Published:** 2025-07-01

**Authors:** Fiona Callanan, Carmel Bradshaw, Teresa Tuohy, Maria Noonan, Sylvia Murphy, Annmarie Grealish

**Affiliations:** 1 School of Nursing and Midwifery, Health Research Institute, University of Limerick, Limerick, Ireland; 2 University Maternity Hospital Limerick, Limerick, Ireland; 3 Kings Florence Nightingale Faculty of Nursing, Midwifery & Palliative Care, King’s College London, London, United Kingdom; Deakin University Faculty of Health, AUSTRALIA

## Abstract

**Introduction:**

There is a growing body of literature on the significance of trauma and abuse in the perinatal period but despite this, trauma exposure and abuse are often not recognised in maternity care settings. Evidence indicates that women experiencing mental distress during the perinatal period are frequently unidentified or inadequately supported. The purpose of this study was to conduct an integrative review on how midwives screen, assess, and respond to women with trauma histories in the perinatal period and to identify the challenges in providing trauma discussions, screening and assessment.

**Methods:**

This integrative review followed Whittemore and Knafl’s five-stage framework as it facilitates the inclusion of different methodological approaches to experimental research. Five electronic databases (PsycINFO, MEDLINE, CINAHL, ASSIA, and Web of Science), reference and citation lists were systematically searched from inception with no date, language or geographical limiters set owing to a dearth of research in this subject area. This review was performed and reported according to the PRISMA guidelines. The findings were analysed and synthesised using narrative synthesis.

**Findings:**

Twenty-two studies met the inclusion criteria and were synthesised using narrative synthesis. Four main themes were identified: 1) Midwives difficulties in asking and discussing interpersonal trauma and abuse and their instinctive use of their observations skills to elicit information; 2) Screening tools to elicit history of interpersonal trauma; 3) Midwife’s response to interpersonal discussions; 4) Training on *‘daring to ask the questions’* and Support on ‘*what should I do now’*.

**Discussion:**

Our findings demonstrate a deficit in trauma discussion, screening and assessment of trauma within the perinatal care, domestic violence being the exception. Interpersonal trauma is a significant public health concern that if left unrecognized may increase morbidity and mortality in both mothers and newborns. This study makes recommendations for urgent streamlined trauma discussions and specific training and supervision on trauma-informed care for all healthcare professions in their perinatal role using a whole systems approach.

## Introduction

The perinatal period which extends from pregnancy to 12 months after childbirth is the most vulnerable time in a woman’s life, as marks a substantial shift in women’s physiological, social and psychological wellbeing [1–4]. During the perinatal period, women’s vulnerability to mental health disorders is increased and is associated with poor maternal and infant outcomes. Prevalence of perinatal mental disorders including depression, anxiety and post-traumatic stress disorder (PTSD) are among the most common morbidities of pregnancy and the postnatal period [3,5–9]. Research suggests that the perinatal period for women is marked by increased vulnerability to interpersonal traumatic events which may be related to complications in pregnancy (e.g., pregnancy loss), labour and delivery or unrelated to their pregnancy (e.g., sexual assault that may have occurred prior to the perinatal period) [10–13]. For the purpose of this review, trauma is defined as an event or circumstance that is perceived as harmful or life-threatening and affects mental, physical, emotional and/or social well-being over the lifespan of the affected person [14]. There are two categories of trauma, Type I trauma is used to identify a single incident or one-off event and Type II or complex trauma which occurs over an extended period, it is often repetitive and anticipated [15].

Although there is a growing body of literature on traumatic experiences and adverse outcomes for women in the perinatal period, this is often not recognised in maternity care settings [10,12,13]. Interpersonal trauma unrelated to pregnancy (e.g., trauma from childhood experiences, sexual assault, rape) and physical trauma (related to childbirth) [1,5,16,17] may be linked to life-threatening or extreme traumatic events, including child maltreatment, intimate partner violence (IPV) and rape, and are highly prevalent among pregnant women [18–23]. Globally, almost one-third (27%) of women aged 15–49 years have experienced some form of physical or sexual violence by their intimate partner [8,22,24–26]. Even higher prevalence rates were reported in a recent review of IPV rates in pregnancy globally ranging from 15.4 to 40% in Portugal, the U.S.A and Australia [27].

The prevalence of women who have experienced childhood sexual abuse has been estimated to be 20% but few women disclose it to healthcare professionals (HCPs) [1,12,13,21,28–30]. History of childhood maltreatment such as physical, sexual, emotional abuse and neglect can have serious consequences on women’s mental health and may include anxiety, depression, PTSD, and posttraumatic stress symptoms (PTSS) [5–10,31]. It also adversely affects physical maternal health, pregnancy outcomes, postpartum maternal mental health, perinatal health risks, bonding, parenting and disrupt family relationships [32–36]. Up to 45.5% of women at 4–6 weeks’ post-childbirth describe their birthing experience as traumatic [37–39]. Maternal trauma exposure is associated with increased prematurity, risk of low birth weight, hypertension, low breastfeeding rates, miscarriage/stillbirth and can disrupt foetal brain development [32,40–43]. Approximately 33% of new mothers have reported the presence of at least three PTSS, primarily anxiety-related, with 9% of these women meeting the Diagnostic and Statistical Manual of Mental Disorders (DSM-V) [44] criteria for PTSD [45–47]. Between six weeks and one-year post-partum, suicide has been reported as the leading direct cause of death [48–51]. Research indicates an association between traumatic experience and adverse outcomes for women resulting in mental health consequences and risk for perinatal depression, anxiety, and PTSD [6,52–58].

With such a high prevalence of interpersonal trauma in the perinatal period it is important for midwives to be aware and informed about best practice when working with women, with the caveat that some women who have experienced interpersonal traumatic event may have completely recovered from their experiences by the time they attend maternity services. HCPs need to be aware that women will respond differently depending on their recovery journey [31]. The Power Threat Meaning Framework [59] offers an alternative to psychiatric diagnosis by identifying patterns of emotional distress. It focuses on what people do and not on the disorders they have, it does not assume pathology but describes coping mechanisms. It is crucial to consider this perspective when working with women who have experienced interpersonal trauma and support them as they navigate their way through their experience.

Promoting women’s health which includes Trauma-Informed care (TIC) is a key priority in perinatal mental health care and is incorporated into the National Health Service [60], Health Service Executive [61,62], U.S. Department of Health and Human Services, Substance Misuse and Mental Health Administration [14,63] and the Mental Health Coordinating Council [64]. TIC takes into account the effect of traumatic experience on current behaviour and can help to minimize re-traumatization during health care encounters [1,65,66]. It helps HCPs move from the concept of “What’s wrong with you?” to “What has happened to you…and how can I support?” [14,63,66]. Screening for trauma is recommended [67–69] and given that maternity services already screen for intimate partner violence and perinatal anxiety and depression it may be feasible to add trauma discussions and PTSD screening to that routine [1,63]. This screening for trauma is imperative for delivery of TIC and it is recommended by numerous professional organizations for HCPs [66–69].

Despite this recommendation, Adverse Childhood Experiences (ACEs) screening has not been widely implemented in antenatal services and current practice in perinatal mental health screening does not usually include discussions focusing on trauma and PTSD [1,12,28,40,63]. It is still rare for assessment to distinguish whether the woman sees her depression and anxiety as related to trauma or not [3,7,22,63,70,71]. It is also important to highlight that some concerns have been raised in relation to universal screening for a history of trauma as this may re-traumatise women, increase the number of referrals for support to healthcare services and potentially stigmatise women [72,73]. However, midwives are ideally positioned to undertake such discussions on trauma given their regular contact with women in the perinatal period [1,12,28,74,75]. Interpersonal traumatic events are often misunderstood in maternity care settings where delayed disclosure impacts on the woman seeking help and support. Women who have experienced interpersonal traumatic events may find the perinatal period difficult in relation to some clinical procedures’ such as positions during labour, intimate examinations, communications with HCPs and loss of control [1,29–31] as these can reactive memories of previous abuse and re-traumatizatise the woman [1].

Given the adverse maternal, perinatal and child health outcomes associated with interpersonal trauma, it is important that midwives can have meaningful trauma discussions with women in the perinatal period. The National Maternity Strategy [76] advises that women who are at risk of developing or experiencing emotional or mental health difficulties in the perinatal period should be identified and recommend a multi-disciplinary approach to assessment and support of these women. Despite women being in regular contact with midwives and other HCPs during the perinatal period, women experiencing mental distress are frequently unidentified or inadequately treated [12,28,74,77]. Many women presenting for care to the maternity services, with a history of interpersonal trauma often do not recognise its effects on their lives. They either do not connect the events to their symptoms or they avoid the topic altogether. This makes it hard to assess and understand someone without knowing about their past [1,12,28,63]. Likewise, midwives may not ask questions that elicit a woman’s history of interpersonal trauma, as they often feel unprepared to address trauma-related issues proactively or may struggle to address traumatic stress effectively within the constraints of the maternity services [1,77,78]. Therefore, this review will examine how midwives can be proactive in trauma-informed screening and assessment, with an aim to enhance the care of affected women.

The NICE guidelines [79] provide all HCPs working with perinatal women a clear remit for assessment of women’s psychological health status. How routine trauma discussions are carried out in the perinatal period remains an important clinical challenge. These trauma discussions are often the first contact between the woman and the midwife, with the woman forming her first impression of maternity services based on their interaction. Thus, how screening is conducted can be as important as the actual information gathered, as it begins the relationship with the woman and may assist or delay disclosures of interpersonal trauma and hence referral for treatment. Assessments for trauma-related experiences require an understanding of perinatal mental health, therefore, instrument selection, trauma-informed screening, assessment tools, and assessment processes will be examined in this review. This paper therefore presents an integrative review conducted to identify how midwives screen, assess, and respond to women with interpersonal trauma in the perinatal period. Specific objectives were to:

1) Identify knowledge, skills and professional behaviours of midwives in discussing and assessing trauma in women during the perinatal period.
2) Identify factors that influence trauma discussions, screening and assessment of women during the perinatal period.
3) Identify challenges in providing trauma-informed discussions, screening, along with specific screening and assessment considerations and guidelines.

## Methods

An integrative literature approach was performed to include experimental and non-experimental research [80] to gain a comprehensive understanding of how midwives are assessing for trauma in the perinatal period. This review reflects the same level of rigor as a systematic review using Cochrane guidance [81], reported in accordance with the PRISMA guidelines [82] and protocol registered on PROSPERO (CRD42022383052). Whittemore and Knafl’s (2005) [80] five-stage integrative review framework was employed within this review which includes: (1) problem identification, (2) literature search, (3) data evaluation, (4) data analysis and (5) presentation.

### Search strategy

A comprehensive search of the Grey literature including Open Grey, Prospero, Joanna Briggs Institute, ProQuest Dissertations and Theses Global, PROSPERO and Cochrane library were searched to ensure no other previous or ongoing reviews was conducted. This search results found a dearth of literature related to how midwives are assessing for trauma in the perinatal period. A Population, Exposure and Outcome (PEO) framework was used to develop the selection of terms used in the search strategy and formulate the following review question: *‘What are midwife’s competencies, knowledge and ability to assess and respond to trauma in women during the perinatal period?’*

Five electronic databases were systematically searched from inception to date of searches (Feb 2024): PsycINFO, MEDLINE, CINAHL, Applied Social Sciences Index and Abstracts (ASSIA) and Web of Science with no date, language or geographical limiters set owing to a deficiency of research in this subject area. Identical searches were used for all databases, using Medical Subject Headings (MeSH) terms and associated text words in the title and abstract, and combined using Boolean operators “OR”/ “AND” to refine, expand and combine the three PEO concepts (See Supplementary file 1 for search terms). Forward and backward citation searches was performed on all retrieved articles by manually hand inspecting the reference lists and tracking citation index of included studies to identify articles for additional studies.

### Eligibility criteria

Studies were included if they: i) reported primary research that sampled a qualified or registered midwife’s competency in assessing or screening for trauma in women during the perinatal period ii) examined the midwife’s competency in assessing for trauma using any mode iii) were primary published studies using quantitative, qualitative, mixed-method design or review studies iv) focused on women over 18 years with self-reported or diagnoses of trauma (Type 1 which is a single/one-off event or Type 2 trauma or complex trauma which occurs over an extended period of time) during the perinatal period. Studies were excluded if they did not meet the outlined PEO framework and were not primary studies, e.g., published in non-peer reviewed journals, dissertations, protocols, conferences, and expert opinion.

### Study selection

All returned citations retrieved from the database were imported into Covidence (https://www.covidence.org) for title/abstract screening and full-text review which removed duplicates across the electronic databases. All titles and abstracts and full-text screening were independently screened involving four reviewers (FC, AG, CB, MN ) against eligibility criteria with any uncertainties and discrepancies resolved collaboratively between all authors.

### Quality assessment

Methodological quality of the primary studies was independently assessed by two reviewers using the Joanna Briggs Institute Critical Appraisal tools (JBI) [83] and the Mixed Method Appraisal tool (MMAT) for the mixed-methods studies [84]. The methodological quality of each study was independently assessed and rated by two reviewers (FC, AG) for rigor, trustworthiness, credibility, relevance and results in accordance with their research methodology (see Supplementary file 2). Discrepancies between the two reviewers were resolved collaboratively through discussion among the remaining authors.

### Data extraction

A Microsoft Excel data extraction form (see Supplementary file 3) was developed and piloted by the review team to extract data from each study including author, year, country, aim, study design, sample size, participant characteristics, data collection/analysis methodology and variables of interest such as type of trauma (type I or II), midwives’ experiences of assessing for trauma within the perinatal period and results (main findings, limitations and future recommendations/gaps in knowledge). The authors developed the coding instructions and guidelines independently in order to reduce the subjectivity of decisions made. Two authors (FC, AG) independently extracted data from the included studies and any questions were resolved through discussion with the other authors to ensure the accuracy of extractions.

### Data analysis

Due to the methodological heterogeneity across the studies in terms of design and outcomes, a narrative synthesis was used to integrate their findings. Narrative synthesis is a procedure for describing, comparing and combining heterogeneous synthesis of findings from multiple studies using text and illustrations to ‘tell a trustworthy story,’ of the findings to meet the review’s aim [85]. Three elements of Popay et al.’s (2006) [85] guidance on narrative synthesis techniques was used to explain the findings of the synthesis: (1) developing a preliminary synthesis; (2) exploring relationships in the data and (3) assessing the robustness of the synthesis. This technique increased the transparency and reliability of the narrative synthesis and the identification of patterns or themes relevant to the research questions to determine midwives’ competencies, knowledge and ability to assess for trauma in women during the perinatal period.

## Results

### Search results

**Fig 1:**
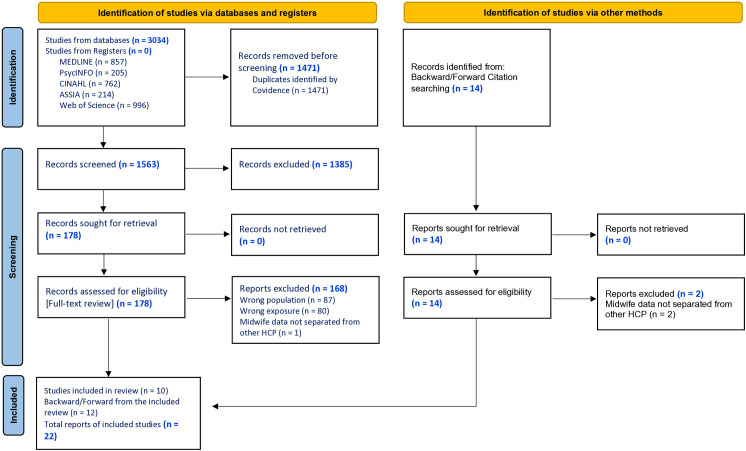
PRISMA 2020 Flowchart.

The database search produced 3034 studies from five databases, 1471 were duplicates, 1563 titles and abstracts were screened, 1385 were excluded with reason, 178 studies were reviewed for full-text screening and 10 studies were identified for inclusion. Hand searching backward and forward citation yielded 14 studies, following full text examination, 12 studies were deemed eligible leaving a total of 22 studies for inclusion in the review. Summary of the systematic search strategy and screening process of eligibility criteria is reported in Fig 1.

### Study characteristics

An overview of the study characteristics is presented in Table 1. All studies were published between 2003–2021 and conducted across ten countries/regions: eight in the U.K, four in Sweden, two in Australia, two in Italy and one each respectively in the Republic of Ireland, South Africa, New Zealand, Netherlands, Norway, and the U.S.A. Most of the included studies were qualitative designs (n = 13) using data from interviews and focus groups, seven were quantitative designs using cross-sectional studies (n = 7) and two were mixed method studies (n = 2).

**Table 1:** Study Characteristics.

Author(s), Year Country	Study Methods	Study Population	Setting	Measurements	Key Findings
Carroll et al. 2018 [74] Ireland	Descriptive quantitative study. Data were collected using an anonymous, self-completed survey.	***Sample size:*** N = 438 midwives ***Participants characteristics:*** Majority of midwives were female (99.8%), employed as midwives in the public health service, educated to primary degree level, and working over 11 years in their role. The distribution across the eight age categories was even (12–15%), with a slightly lower proportion aged 20–24 (5.6%). About 66% of midwives worked in the areas of antenatal care, labour and postnatal care. A further quarter (24.3%, n = 106) worked in other areas. It is estimated that 24.8% of midwives nationally responded. ***Type of trauma (Type I or II):*** Study based on Midwives experiences of women with any perinatal mental health concern including trauma (type I or II).	All registered midwives with the Nursing and Midwifery Board of Ireland (NMBI) and employed either full-time or part-time in 12 of the 19 maternity services in the Republic of Ireland were targeted for inclusion in the study.	Knowledge of perinatal mental health was rated on a scale from 1 (not at all knowledgeable) to 5 (very knowledgeable). Questioning/Asking about trauma – On opening a discussion with women about various topics, self-reported skill was rated below the midpoint of the scale for sexual abuse (M = 2.15, SD = 1.02) and intimate partner violence. Skill was rated higher on providing support to women who were traumatised by their birth experience (M = 3.50, SD = 0.98) and who were emotionally distressed (M = 3.22, SD = 0.99). Overall skill midwives rated below the midpoint of the scale (M = 4.48, SD = 1.82).	Midwives in this study tended towards asking women they considered to have mental health risk factors about experiences of mood disorders, anxiety and past trauma rather than asking all women. Selective screening practices based solely on clinical judgement can result in at-risk women being missed. This may also be related to the fact that a clear majority of midwives in this study did not report using screening tools to assess mental health. Perceived skill in discussing issues with women were low (sexual abuse, psychosis, intimate partner violence, eating disorders and self-harm/suicidal thoughts) were also areas that were somewhat neglected within practice, with significant number of midwives never asking women about sexual abuse/sexual violence, intimate partner violence, experience of eating disorders or psychosis. This study findings suggest a need for a more comprehensive approach to the education of midwives on the range of perinatal mental health problems that may be experienced by women.
McKenzie-McHarg et al. 2014 [86] U.K	Qualitative study. A pilot evaluation of the pink sticker system. 1)Focus Group (n = 4) 2) In-depth discussions telephone/in person conversation (n = 4) 3) Postnatal followed up with their psychologist (n = 49) 4)Psychology referral database at Warwick hospital was examined to establish the proportions of perinatal referrals with a diagnosis of birth trauma or PTSD	***Sample size:*** Interviews and clinical feedback from women (group, telephone and postnatal follow-up, n = 53). ***Participants characteristics:*** Majority The midwives ranged in seniority from at least 2 years qualified to very senior, and had all cared for a number of women who had presented to the labour suite with a pink sticker on their notes within the last year. ***Type of trauma (Type I or II):*** Type 1 and Type 11. Typical examples of women being given a pink sticker included women who had experienced a previous traumatic birth, had a needle phobia, had lost a baby, had a fear of childbirth, or a history of abuse.	Data was gathered from the hospital during antenatal, intrapartum and postnatal period.	No screening tools used.	Overall, the pink sticker psychology alert system was perceived positively by both women and midwifery staff. Only 15% of women reported disadvantages of the system, and all these women also reported advantages. It is likely that no wording or different wording on the stickers would address those issues. Main disadvantages mentioned were that some women were referred too late to receive a pink sticker and there were some impractical requests made on pink sticker summaries. Psychology staff using pink stickers will be given joint training from senior psychologists and senior midwives on the appropriateness of various requests to ensure that those requests made on pink sticker summaries are feasible. The overall reduction in referrals for traumatic birth from 2009 to 2013 was 44%. Crucially, no woman birthing with a pink sticker developed trauma symptoms which could be attributed to poor communication or negative perceptions of care during her delivery experience.
Finnbogadóttir & Dykes 2012 [87] Sweden	An inductive qualitative design was chosen. The data collection method consisted of focus group interviews.	***Sample size:*** N = 16 midwives ***Participants characteristics:*** The particular working area experience of the recruited midwives varied within the group and included activities such as working with women who have a ‘fear of birth’, or ‘substance abusers’, or ‘birth’, ‘postpartum care’ or ‘sexual health guidance’. The mean working experience was 22 (min 4–max 36) years. ***Type of trauma (Type I or II):*** Type II Domestic Abuse	Four focus groups were assembled. All but one of the midwives was working in ANC units connected to two university hospitals in southern Sweden.	No measures or screening tools utilized.	The midwives expressed clearly that they felt insufficient in their approach and care of the mother and thus, insufficient also with regard to the unborn baby. Currently, the midwives have no written guidelines, plans of action and have insignificant or non-existent support from the employer. In the present study the midwives regarded the documentation of abuse not only as a security problem at the ANC, but also as an infringement on the woman’s personal integrity. Avoidance of questions concerning the experience of violence during pregnancy may be regarded as a failing not only with regard to the pregnant woman but also with regard to the unprotected and unborn baby. Midwives need better working conditions and support to have possibilities to take care of this complicated topic. Although midwives were aware of the need to address domestic violence during pregnancy, there appears to be a number of obstacles that need to be overcome before the introduction of routine enquiry.
Rollans et al. 2013 [88] Australia	This qualitative ethnographic study describes the content and process of psychosocial assessment and depression screening undertaken by midwives in the antenatal booking visit.	***Sample size:*** N = 16 midwives (M) and N = 2 two student midwives (SM) undertook psychosocial assessment and depression screening ***Participants characteristics:*** The average years of experience of the 16 midwives were five years and 12 of these midwives had worked an average of three years in the antenatal clinic (AC). Three of the participants were working in a midwifery group practice ***Type of trauma (Type I or II):*** Type 11. Questions used in psychosocial assessment included ACE’s, Domestic violence, couple’s relationship problems, history of anxiety, depression or mental health problems.	Midwives at the antenatal booking visit in two maternity units in New South Wales(NSW), Australia.	This paper reports on the content and process of antenatal psychosocial assessment & depression screening undertaken by midwives at the booking visit. A structured observation tool and field notes were used to record observations of the assessment and screening process. The observation was developed for this study was called: 4D&4R. The 4 D’s (introduce, deliver, deal and debrief) were used to record details about the overall approach taken by midwives to the psychosocial assessment and screening inc how midwives introduce the psychosocial questions and depression screening tool	Midwives were observed using a range of skills when undertaking psychosocial assessment including empathetic responding, however, modification of questions may reflect a level of discomfort on the part of the midwife in asking sensitive questions and may impact on the integrity of the assessment. Further training and support is required to ‘fine tune’ the process of assessment and better respond to disclosure of sensitive information. Implications for practice: midwives require organisational support for ongoing training and clinical supervision to effectively undertake routine psychosocial assessment.
Baird et al. 2013 [89] U.K.	A multimethod approach was adopted, using a follow-up survey and focus groups. Collecting the views and experiences of midwives in relation to routine enquiry for domestic abuse.	***Sample size:*** N = 58 participating midwives completed a 54-item questionnaire. Midwives were asked to reflect on the content and learning outcomes of the original training and subsequent mandatory follow-up study days. Perceptions around practice were explored in more depth within the focus group interviews ***Participants characteristics:*** Three (6%) of the participating midwives had been qualified for less than five years, 15 (29%) qualified between five and 15 years, three (6%) between 15 and 20 years and 31 (60%) more than 20 years. It is estimated that 36 (73%) of the 2010 respondents took part in the original study; of those who responded 14 (27%) held a certificate, another 15 (29%) held a diploma and a further 15 (29%) held an honours degree. 23 (44%) covered some form of domestic violence education in their pre-registration training; 37 (71%) of the midwife population had attended update sessions as part of their mandatory programme either annually or biannually. 49 (96%) of the midwives taking part self-reported some level of professional experience in dealing with domestic abuse, with four (7%) reporting a great deal of experience, 19 (37%) reporting a moderate amount, and 26 (51%) reporting a minimal amount. ***Type of trauma (Type I or II):*** Type 11 Domestic Violence	The final sample included both the original community midwives who had taken part in 2004/2005 and those who had been subsequently recruited or had transferred into the Trust. 36(73%) of the 2010 respondents took part in the original study. Of these, a purposive sample of 11 took part in two focus group interviews.	No Tool	This is the first UK based follow-up study to evaluate the outcomes of a maternity educational intervention in domestic violence enquiry five years on from its introduction. Outcomes from the study included midwife’s abilities to ask women about domestic abuse, feeling supported and appropriate referral. Five years on, the statistical evidence suggests that the skills, knowledge and confidence associated with antenatal enquiry for domestic abuse developed through the 2004/2005 BPDVP programmes have been maintained, with the support of mandatory training. However, it was midwife’s attitudes in relation to their role in domestic violence that had changed significantly, with all (100%) of those surveyed reporting that enquiry was now considered a fundamental part of their role. The findings from this study demonstrate that across the cohort there was a statistically significant increase in self-reported confidence in asking women about domestic abuse. In addition, there was a statistically significant increase in the degree of self-reported knowledge of how to deal with a disclosure of domestic violence when comparing the 2010 data with 2005 data.
Di Giacomo et al. 2017 [90] Italy	A cross-sectional study. This follow up study used a questionnaire. Fifty-one (63 7%) questionnaires were analysed and were compared to 44 of the survey of 2008.	***Sample size:*** N = 56 health care operators completed the questionnaire. ***Participants characteristics:*** Forty-one (80.4%) participants were female, 35 (68.7%) were aged 40 and under, 31 (60.8%) were nurses, and 20 (39.2%) were midwives. Twenty-four (47.1%) nurses were working in the emergency room. Fifty-six (70%) healthcare operators completed the questionnaire. ***Type of trauma (Type I or II):*** Type 11 Domestic Violence	This study was conducted in the departments of the Health Company of Rimini, which is in charge of receiving women who suffered violence: emergency room, (two department), obstetrics emergency room, gynaecology and Department of Gynaecology and Family Counselling (four wards).	No Tool	Of the respondents, 51 (80 4%) have taken care of women who suffered violence, and 25 (49%) believe they can detect violence. The relational/communicative approach presents the most difficulty, and all the operators believe they need more knowledge. The number of operators who suggest women be observed in an emergency room and file a complaint or who primarily consider listening to women has decreased. A tendency to ‘blame’ women, although decreasing, persists; it is higher among male nurses and, in general, among male operators. Nurses and midwives, most of whom are women, are in a unique position to screen, during every visit, every woman who has suffered abuse; they can intervene properly and conduct further research by using the referenced experiences of abused women to reduce domestic violence.
Salomonsson et al. 2010 [91] Sweden	A qualitative study, Phenomenographic approach – analysis only.	***Sample size:*** N = 21 midwives ***Participants characteristics:*** Midwives aged 27–63 years (median 52). Professional experience varied from 3 to 38 years (median19). Expressed in years, the participants’ experience was spread as follows: antenatal clinics including family planning, range 0–17 years (median 6); labour ward, range 0–30 years (median5); and pre-and postpartum ward, range 0–35 years (median5). ***Type of trauma (Type I or II):*** Type 1 Traumatic Birth	Four hospitals with varying levels of care were included in the study (in two counties in southern Sweden, one at a university hospital, two at central county hospitals and one at a county hospital)	No formal assessment measures/screening tools used. (observation)The identification of FOC occurs during pregnancy, but it can also take place during the labour process or postpartum. The women present their fear to the midwife, with or without being asked. Women may not openly communicate their FOC and expressed it in other ways such as ‘denying their pregnancy. others, the fear is embedded in numerous pregnancy-related symptoms: the midwives term ‘unclarified’ fear, which means that the woman	FOC can hinder a normal experience of pregnancy, the labour process and the transition to parenthood. Working with women with FOC is emotionally demanding and time consuming. FOC should be identified early in the pregnancy or even before. Women with FOC need individual care and preparation for labour and delivery. The midwives had no problem talking about the subject, and all took an active part in the discussions. In Sweden, most maternal care centers have formed ‘FOC teams’ to which women with FOC can be referred for support. The midwives pointed out the importance of identifying women with FOC and of supplying professional support adjusted to individual needs. Processing fear during pregnancy and preparation for childbirth are regarded as central elements in a midwife’s work with women with FOC.
Shamu et al. 2013 [92] South Africa	An exploratory qualitative study.	***Sample size:*** N = 6 midwives ***Participants characteristics:*** At each of the six clinics, one senior nurse midwife (sister-in-charge) in charge of the maternity clinic was purposively selected to participate in the interviews. Age of midwives ranged between 40 and 60 years. ***Type of trauma (Type I or II):*** Type 11 Domestic Violence of IPV	The setting of the study is within six public antenatal care clinics located in low-income residential areas in Harare.	No screening tools used to assess for trauma.	Lack of education and skills to screen for violence, the health system’s limited human resources capacity and its failure to promote screening, and role conflicts reflecting a deep ambivalence over whether IPV is a health problem, a social problem or simply ‘normal’ were identified as the major obstacles to responding to IPV. Institutional reform that will lead to training midwives and integrating IPV responses in antenatal care services could help in addressing IPV in antenatal care in culturally appropriate and sensitive ways.
Mortimore et al. 2021 [93] U.K.	The project piloted a service improvement with a defined cohort of women and their partners. A pragmatic mixed-methods approach was selected.	***Sample size:*** N = 8 midwives ***Participants characteristics:*** No data ***Type of trauma (Type I or II):*** Type 11 ACE	Gloucestershire ANC	Midwives introduced the ACEs screening tool to booking women and their partners (if present). Following completion, a discussion took place to enable the ecomap to be populated and any support needs identified, leading to development of a mutually agreed, individualised plan for the family. The ACE or ACEs identified were used to initiate discussion with a focus on development of support plans. M card; a small folded leaflet the size of a credit card given to women with the booking pack to explain the meaning of adverse childhood experiences (ACEs) and the five ways to wellbeing, a self-help aid to reduce the impact of ACEs. As well as being an aid to parents, this tool was used by midwives to help start the conversation about ACE.	The use of the ACEs screening tool successfully identified ACEs which would otherwise not have been known using the previous antenatal booking questions. The bespoke tool kit was well-received by women, their partners and professionals. Identification and discussion of ACEs enabled appropriate support to be offered. The rates of disclosure for one or more ACE were 57%, higher than that described in the general population. The toolkit was found to be effective in identifying ACEs for parents and their unborn baby. Midwives are well-placed to support women and families to identify risk, build parental resilience and strengthen parenting capacity. This could help protect future generations from a cycle of adversity.
Stenson et al. 2005 [94] Sweden	Qualitative study design using focus-group discussions in antenatal care in a city in south-central Sweden.	***Sample size:*** N = 21 midwives took part in one of five interviews (two to six participants each). ***Participants characteristics:*** Participants were 42–62 years old (median 54 years), had been midwives for 8–39 years (median 26 years) and had been working at antenatal clinics in the county for 0.5–26 years (median 12 years). ***Type of trauma (Type I or II):*** Type 11 Domestic Violence	Seven antenatal clinics in a city of 180,000 inhabitants in south-central Sweden	The questions were derived from the Abuse Assessment Screen (Parker and McFarlane, 1991). Questions asked as part of the psychosocial assessment.	To implement routine questioning about violence is not a one off event. The management must give unflagging support (e.g., by establishing and monitoring routines, and by providing training, counselling and feedback). The midwives emphasised the importance of training, easy access to support for abused women, and personal counselling for midwives. They described their role as raising awareness of the problem of male violence, reducing the shame of being abused, informing, giving emotional support and mediating help. in order to ensure that the establishment of experience of violence is a routine enquiry in antenatal care, midwives must have a reasonable opportunity of carrying out such questioning. A routine that offers each woman a private consultation will ease the questioning and save time and distress.
Jackson et al. 2009 [95] U.K.	A mixed methods approach was used using a survey (self-administered postal questionnaire). The questionnaire included fixed response and open-ended questions.	***Sample size:*** N = 372 midwives, representing a 76% response rate. ***Participants characteristics:*** Midwives from all areas of midwifery care were included, e.g., labour suite, antenatal clinics, antenatal and postnatal wards, and community, to ensure that all midwives’ knowledge and attitudes of the topic matter were presented. Midwives years of experience 0–5 years 109/29%, 6–10 years 82/22%, 11–15 years 86/23%, 16 + years 94/25%, hospital based Midwives 241/65%, community based Midwives 130/35%. ***Type of trauma (Type I or II):*** Type 11 Sexual Abuse	The sample group selected was from different trusts within the East and West Midlands (n = 489). The questionnaire was piloted twice	No Tool	The majority of midwives in the sample group did not feel adequately prepared to deal with a disclosure of sexual abuse (n = 207, 56%), with a further 109 (29%) midwives being ‘unsure’ if they could deal with such a disclosure. Community midwives rated themselves as more able to deal with disclosures of sexual abuse compared with hospital-based midwives (w2 = 4.044, df ¼ 1, p = 0.044). It is possible that midwives who have been inadequately prepared for dealing with such disclosures may give inappropriate advice, and may inadvertently compound the feelings of powerlessness that women have experienced. Aspects of sexuality and, in particular, caring for women who have been sexually abused should be included in all pre-registration curricula, and post-registration education should include this topic to either update midwives or introduce these topics to qualified midwives.
Lazenbatt et al. 2009 [96] Northern Ireland	This quantitative study was conducted between December 2002 and August 2003 using a postal questionnaire. The Midwives’ Knowledge and Attitudes to Domestic Violence Scale was developed and validated for the purposes of this study.	***Sample size:*** N = 488 midwives ***Participants characteristics:*** Midwives based in a hospital setting accounted for 80% of the cohort, with 39% employed full-time, and 57% over 40 years of age. Significantly more community midwives (65%) were employed full-time [continuity corrected w2 = 20.4, degrees of freedom (df) = 1, po0.001], and significantly more community midwives (71%) were 40 years of age (continuity corrected w2 = 5.64, df = 1, po0.05). ***Type of trauma (Type I or II):*** Type 11 Domestic Violence	A letter was sent to all registered NI midwives working in seven locations (n = 983) inviting them to participate in this study. The locations included five hospital and community settings.	No Tool	Although the main findings show that almost all midwives (92%) felt that they had a significant role to play in responding to DV in their client group, data illustrate that only half of those participating were in favor of routine screening, and only 38% of the cohort had actually made an enquiry. Worryingly, 79% of hospital and 72% of community midwives were unsure or not confident about identifying DV in practice. The paper highlights that midwives per se identify and respond to a fraction of the cases of DV in pregnancy due to a lack of confidence and up to-date knowledge and education. This finding reinforces the need for both hospital and community midwives to gain further confidence and an understanding of the psychosocial issues surrounding DV. Limited confidence is often underpinned by lack of education.
Mauri et al. 2015 [97] Italy	A qualitative phenomenological-hermeneutic study design.	***Sample size:*** N = 15 midwives ***Participants characteristics:*** The average age of the surveyed midwives was 37 years (range 22–55), and their average working experience was 14 years (range eight months to 35 years). ***Type of trauma (Type I or II):*** Type 11 Domestic Violence	Midwives working in the local health district of Monza and Brianza (northern Italy): four were practicing in the community, and eleven worked at Monza’s San Gerardo tertiary hospital.	No Tool	Given the prevalence of domestic violence during pregnancy and its negative consequences on maternal and foetal health, it seems to be essential to implement first-level and continuing education for midwives on the subject. Midwives acknowledge their crucial role in identifying and managing domestic violence but are still unprepared to do so and indicate various barriers that need to be overcome. There is a need to implement basic university education on the subject and provide specific professional training.
Eustace et al. 2016 [98] Australia	A qualitative descriptive design was used	***Sample size:*** N = 15 midwives ***Participants characteristics:*** The majority of participants were female, worked in the public health system, in a range of different models of maternity care including shared care based in an obstetric antenatal clinic, midwifery led clinics and midwifery caseload practice and practiced in tertiary units, rural and remote hospitals and community. Midwives female n = 20/95.2, male n = 1/4.7%. Queensland n = 5/28.8%, New South Wales n = 4/19.0%, Victoria n = 4/19%, Tasmania n = 0, Western Australia n = 2/9.5%, South Australia n = 2/9.5%, Australian Capital Territory n = 2/9.5% and Northern Territory n = 2/9.5%. Public n = 19/90.5%, Private n = 2/95%. ***Type of trauma (Type I or II):*** Type 11 IPV/Domestic Violence	Inclusion criteria were midwives engaged in antenatal service provision within Australia.	Participants described different ways of screening women for IPV. Some midwives reported asking all women directly at the booking interview, whereas others identified the use of a self-report screening tool either written or online that women completed at booking. No detail given on these tools. Midwives working in fragmented models of care highlighted frustration with a system that did not allow time to build rapport with women before asking sensitive questions.	Routine enquiry about domestic violence is a valuable and important midwifery role. Midwives described frustration and fear when women disclosed domestic violence. The perceived level of support from health services is varied according to practice contexts and needs to be improved. All participants described receiving very little training in preparation for asking women about IPV. The majority of midwives identified minimal education on IPV either during undergraduate programs or after employment as a registered midwife. The majority of participants however reported receiving no initial or ongoing education on IPV. Recent research on Australian midwives’ knowledge about IPV and practices confirmed previous research from the United Kingdom about the consistent association between higher levels of knowledge and frequency of asking women about IPV. Midwives who worked in practice environments which incorporated training on IPV and had clear referral pathways reported feeling more confident in dealing with positive disclosures. Continuity of care was identified as a positive enabler for routine enquiry. Midwives valued both the trusting relationship and the opportunity to find the right time to discuss IPV with women.
Ali et al. 2021 [99] U.K.	Cross-sectional survey of preregistration nursing and midwifery students and registered nurses and midwives.	***Sample size:*** N = 326 midwives ***Participants characteristics:*** Most of the participants were midwives (n = 326, 42.39%), followed by nurses (n = 238, 30.95%), and nursing and midwifery students (n = 205, 26.66%). Most participants (n = 657, 25.44%) identified themselves as White English/Welsh/Scottish/Northern Irish/British. The mean age was 40.30 11.99, with most of the participants between the ages of 46–55 (n = 326, 42.39%). The mean years of experience was 14.01 (SD 13.06), and the mean hours of training on DV was 11.27 (SD 42.09) (missing data = 98). ***Type of trauma (Type I or II):*** Type 11 Domestic Violence	The study participants were registered nurses and registered midwives as well as preregistration nursing and midwifery students, studying and working in England and Wales. To collect data from registered nurses and midwives, the Royal College of Nursing (RCN) and Royal College of Midwifery (RCM) were approached and requested to distribute survey electronically to its members.	No Screening tool.	Over all, the study findings indicate that nurses, midwives and students find it challenging to manage cases of IPV due to limited knowledge and preparedness. Nurses and midwives recognized the need to manage cases of IPV effectively, but they lacked necessary resources and training. Therefore, a need exists for greater training, resources, and support for nurses, midwives, and students to manage cases of IPV. The training should focus on recognizing the cases of IPV, its health impact on patients, screening for and communicating with the individuals who experienced IPV, and the care modalities to enhance person-centred trauma informed care (Alshammari et al., 2018; Alvarez et al., 2018). Nurses and midwives also used the learning from past experiences of managing and experiencing IPV to manage new cases. The study findings reiterate the need for greater training and education of nursing and midwifery professionals regarding IPV screening, management and care modalities. Greater emphasis should be placed on the education of students so that they are better prepared to handle IPV in clinical practice. Midwives had more positive attitudes compared with nurses towards women experiencing intimate partner violence.
Lazenbatt and Thompson-Cree, 2009 [100] Northern Ireland	Quantitative study design using a validated questionnaire.	***Sample size:*** N = 488 midwives ***Participants characteristics:*** Midwives based in a hospital setting accounted for 80% of the cohort, with 39% employed full-time, and 57% over 40 years of age. Significantly, more community midwives (65%) were employed full-time (continuity corrected χ2 = 20.4, d.f. = 1, P < 0.001), and significantly more community midwives (69%) were over > 40 years of age (continuity corrected χ2 = 5.64, d.f. = 1, P < 0.05). ***Type of trauma (Type I or II):*** Type 11 Domestic Violence	All registered NI midwives working in seven locations (n = 983) were invited by letter to participate in this study. The locations included five hospitals, four of which were integrated to community and two community settings.	No formal screening tool used. Only 13% of the sample actually asked a woman a direct question about DV.	The study suggests that midwives need training on how to interact with abused mothers using non-coercive, supportive and empowering mechanisms. Many women may not spontaneously disclose the issues of child or domestic abuse in their lives, but often respond honestly to a sensitively asked question. This issue is important as only 13% of the sample actually asked a woman a direct question about DV. The present study shows that almost all midwives (92%) felt they had a role to play in responding to DV, and just over half felt that all women should be screened. However, only 28% of the sample had asked a woman about DV, and only 13% had asked a direct question concerning abuse. Perceived role in relation to identifying suspicious or definite cases of CA: 96% of both hospital and community midwives believed they have a significant role in identifying a child’s risk factors during their mother’s pregnancy and post-natal period. A high proportion of both groups acknowledged a link between domestic and CA, 96% felt midwives had a significant role in identifying CA and 87% reported a willingness to get involved in addressing CA issues. Findings suggest that lack of education and training was a problem as only a quarter of hospital-based midwives reported to have received training on DV and 40% on CA. This was significantly less than that received by community midwives, as 57% received training on DV, and 62% on CA. The study suggests that midwives need training on how to interact with abused mothers using non-coercive, supportive and empowering mechanisms. Many women may not spontaneously disclose the issues of child or domestic abuse in their lives, but often respond honestly to a sensitively asked question.
Mezey et al. 2003 [101] U.K.	Mixed Methods approach adapted. Focus groups, semi-structured interviews and a questionnaire was used to collect information.	***Sample size:*** N = 28 midwives ***Participants characteristics:*** Of the 116 trained midwives, 109 (94%) were trained in time for the booking screening phase, of whom 81 (74%)participated in the booking screen. At the 34 week screening phase, 27 (23%) of the 116 trained midwives participated. Of the 57 midwives who were trained and who were working on community teams during the postnatal screening phase, 23 (40%) participated. ***Type of trauma (Type I or II):*** Type 11 Domestic Violence	One hundred and forty-five midwives from 8 hospital teams, 10 community teams, specialist midwives and mid wifery managers were invited to attend a training session, of whom 116 (80%) attended.	Midwives carried out screening in hospital and community antenatal clinics and women’s homes on three occasions: at booking, at 34 weeks and once during the postpartum (within 10 days) using a variation of the Abuse Assessment Screen.	Midwives felt that domestic violence was an important issue to be addressed. However, practical and personal difficulties were encountered during the study. These included: time constraints, lack of confidential time, safety issues, staff shortages, low staff morale and midwives’ personal experiences of domestic violence. The study produced a raised awareness about domestic violence within the maternity unit. There was considerable support for the idea of a specialist domestic violence Midwife. Routine enquiry for domestic violence cannot be implemented effectively without ensuring that in-depth training, resources, staff support and policies to ensure that screening can be conducted safely and confidentially are in place. Most midwives were in favour of a specialist domestic violence midwife to co-ordinate training, act as a support system for midwives to discuss their own feelings and to which they could refer very distressed women
Lauti et al. 2008 [102] New Zealand	Qualitative methodology. Focus groups and semi-structured interviews with midwives and obstetricians were conducted, recorded and analysed.	***Sample size:*** N = 5 midwives ***Participants characteristics:*** Midwife participants included two hospital midwives, two independent midwives in community-based practice and one hospital midwife who had also worked independently. The five Midwives had an average of 9.8 years in midwifery practice, and all but one reported attending courses about family violence. ***Type of trauma (Type I or II):*** Type 11 Domestic Violence	All Midwives and Obstetricians at Dunedin Public Hospital	No tool	Maternity health professionals in the locale studied have significant issues and difficulties in the identification and management of family violence. These need to be addressed in training programmes and guidelines to improve patient outcomes, and to provide support and safety for clinicians. The emphasis of family violence identification has been on current abuse, but identification of historic abuse may also be important and relevant, especially if there are unresolved issues. Debriefing after managing emotionally difficult cases was considered beneficial by both midwives and obstetricians in reducing adverse impact. Practitioners need to feel safe and supported in their practice with adequate support networks readily accessible. Practitioner safety and debriefing measures should be included in guidelines, training and practice.
Hindin, 2006 [103] U.S.A.	Qualitative study using interviews to collect data.	***Sample size:*** N = 8 CNM’s ***Participants characteristics:*** The CNMs are all employed in full-time clinical practice, are female, and Caucasian. They ranged in age from 30 to 56, with a mean age of 41 years. Their clinical practice experience ranged from 6 months to 32 years, with a mean of 8.6 years of clinical practice. Six CNMs were educated at the master’s degree level, whereas two were educated at the postmaster’s certificate level. Seven have received domestic violence education, and one has not. They received their educations at seven different universities throughout the country. They reported working in both private practice (three midwives) and public clinics (five midwives) and working with 13 different cultural groups of women. ***Type of trauma (Type I or II):*** Type 11 Domestic Violence	The CNMs are members of ACNM and live and practice in the Midwest.	None of the participants used a standardized screening assessment tool, such as the Abuse Assessment Screen, but they adapted their questions based on content in this tool and their individual clinical style. However, they all depend on clinical suspicions or that “gut reaction” throughout pregnancy, during labor, and in the postpartum period in place of routine screening to alert them to potential abusive situations.	The findings demonstrate that the midwives were inconsistent in their intimate partner violence-screening practice during pregnancy and increase or decrease screening in response to a woman’s cultural background. The results of this study indicate that the midwives are concerned, interested, and knowledgeable about intimate partner violence screening, and the participants in this study do conduct screening. However, they are inconsistent in their adherence to universal screening clinical practice and ACOG clinical guidelines for domestic violence. The participants in this study adjusted the recommended number of screenings (four) based on subjective assessment of each woman and her partner. The midwives screened less than half of the recommended time and were influenced by a woman’s culture to increase or decrease their screening frequency.
Nyberg et al. 2010 [104] Sweden	A qualitative research method was used because the purpose was to describe midwives’ experience of encountering women with posttraumatic stress symptoms after childbirth.	***Sample size:*** N = 8 midwives ***Participants characteristics:*** Some of the participating midwives had special training in posttraumatic stress counselling and all of them had more than 12 years of experience in the midwifery profession. ***Type of trauma (Type I or II):*** Type 1 Traumatic Birth	Inclusion criteria of having an experience of working in specialized clinics for women with posttraumatic stress symptoms. In Sweden there are clinics for women with fear of childbirth and posttraumatic stress symptoms after childbirth.	No Tool	It is shown that the midwives’ support during childbirth has positive long-term effect on the woman’s memory of childbirth. Fear of childbirth should be identified in an early stage/pregnancy to allow early treatment on the right level of care. The entire obstetric care process should focus on supporting the women to feel safe, respected, well informed, and involved. Training of midwives is necessary to increase awareness of the cause of women’s fear of childbirth. A woman’s need to be respected and listened to seems to be one of the most important health care issues in the midwives’ encounter with women.
de Vries et al. 2020 [105] The Netherlands	A cross-sectional study was performed amongst midwives who work in community practices and hospitals in the Netherlands with the use of a questionnaire.	***Sample size:*** N = 217 midwives ***Participants characteristics:*** Demographics: Female n = 214/98.6%, male n = 3/ 1.4%, Age: 20–24 n = 4/1.8%, 25–34 n = 73/33.6%, 35–44 n = 73/33.6%, 45–54 n = 42/19.4%, 55–64 n = 25/11.5%. Position: Community Midwife (practice owner) n = 78/35.9%, Community Midwife (paid employee) n = 15/ 6.9%, Community Midwife (substitute) n = 11/5.1%, Clinical Midwife (Secondary Care) n = 89/41.0%, Clinical Midwife (tertiatory care) n = 14/6.5%, Researcher/Teacher/Sonographer n = 4/1.8%, Retired n = 1/0.5%, Non-Practicing/Management Position n = 5/2.3%. ***Type of trauma (Type I or II):*** Type 1 Traumatic Birth	In April 2016, Dutch midwives associated with the Committee of Clinical Midwives of the Dutch Society Obstetrics and Gynaecology (NVOG) (199 members), Talmor B.V. (1100 members), midwifery practices in the Leiden/the Hague region (181 community midwives) and OLVG hospitals in Amsterdam (42 clinical midwives) received an invitation by e-mail to participate in the study. Additionally, an announcement was made in the newsletter of the Royal Dutch Organisation of Midwives (KNOV) (3561 members) requesting midwives to participate.	A total of 203 midwives answered the question whether or not they actively reviewed the presence of FoC during prenatal check-ups. In 57.6% (N = 117/203) of the cases midwives always asked and 40.9% (N = 83/203) of the participants only addressed it in specific situations, depending on history and signals or risk factors of FoC. Midwives were asked whether they were used to asking women about their birth experience in the postpartum period; 81.1% (N = 150/185) confirmed to do so directly or within one week after the birth (100%, Ncommunity = 78/78 vs. 66.7%, Nclinical = 68/102, p =<0.001). During the six weeks’ postpartum check-up all midwives confirmed asking women about their birth experiences. No formal screening tool used by midwives.	Women suffering from fear of childbirth and postpartum posttraumatic stress disorder are often not recognised by health care professionals. Midwives should acquire more in depth knowledge of fear of childbirth and postpartum posttraumatic stress disorder. This can be achieved by including the two conditions in the program of midwifery education. The knowledge and awareness of FoC was greater than the knowledge and awareness of PTSD since more midwives were able to answer knowledge questions on FoC. Knowledge about FoC and PTSD can be obtained and preserved in various ways, starting with students of Midwifery. In the Netherlands, current students in programs of midwifery gain substantial knowledge on perinatal mental health disorders, including FoC and postpartum PTSD. This, however, is a recent addition to the curriculum, largely because research and clinical awareness about these conditions originates from the last two decades. In our study many of the participants were well informed on the symptomatology of FoC. As expected, midwives primarily identified women with FoC through psychological symptoms such as anxiety and mood changes, as well as new physical complaints such as headaches and sleeping problems. An important finding was that in daily practice, most midwives also suspect FoC based on medically related signs, such as requests for caesarean section. Although midwives were aware that psychological status and history of women is an important risk factor for developing FoC, a higher recognition rate was expected. Ensuring that (future) midwives gain substantive knowledge on perinatal mental health disorders such as FoC and postpartum PTSD is a crucial the task of programs of midwifery education.
Fenne Fredriksen et al. 2021 [106] Norway	Qualitative research design with semi-structured interviews.	***Sample size:*** N = 10 midwives ***Participants characteristics:*** The participants were recruited through a liaison meeting of mid wives working in primary care. 10 Midwives in total participated in the study. The age range was 32–61 years, with experience in antenatal care from 0.5 to 17 years. ***Type of trauma (Type I or II):*** Type 1 Domestic Violence	Midwives from eight Norwegian municipalities were included. Data collected through semi-structured interviews. An interview guide was prepared with seven open-ended questions. The interview was conducted by one of the co-authors, and another author transcribed the tapes. Tape recordings were made of the interviews and these were transcribed verbatim.	No Tool	The amount and type of training midwives received from the start varied considerably across the different workplaces. One midwife described how there was very little training on the issue when she started working. The findings show that the screening involves more than simply asking a question. It was difficult to uncover ongoing violence, but they had been able to reveal numerous incidents of past violence. While they were marked by this experience, they stressed the importance of daring to ask, having the mettle to listen to the response and dealing with the situation. Asking about violence became easier with time, but support from colleagues was important whenever difficult cases arose. The results show that the midwives had received different types of training. This study reveals a need for skills enhancement with respect to violence among healthcare personnel as well as in society in general. Another important finding was that some midwives felt that their own safety is compromised in certain situations when they uncover violence.

The included studies reported a combined total of 2615 midwives involved in the care of women with history of trauma. Study sample sizes ranged from 4 to 488 midwives, the mean age reported from eleven studies was 40 years old [74,90–92,94,96,97,99,100,103,105,106]. Clinical experience in midwifery ranged between 6 months to 32 years, with the average amount of clinical experience reported to be over ten years in thirteen of the studies [74,86–89,91,94,95,97,99,102–104,106]. All midwives in the included studies were women (n = 2615). Ethnicity was reported by only two studies, with both studies classifying most of the sample as Caucasian or white English/Welsh/Northern Irish/British [99,103].

Many of the studies (n = 14) focused on midwife’s experiences of caring for women with a history of domestic violence [87–90,94,96–103,106] and the remainder on sexual abuse [95], Adverse Childhood Experience (ACE) [93], both ACE and domestic violence [74,86,88] and traumatic birth/PTSD [91,104,105].

All studies (n = 22) recruited midwives (n = 2615) from maternity services. In six studies midwives conducted assessments in antenatal clinics in hospital settings [87,88,92,94,104,106] with another five studies midwives collected data in antenatal, intrapartum and postnatal settings in a hospital-based setting [74,86,90,91,95]. Seven studies reported data from midwives both in hospital and community settings [96–98,100–102,105] and the final two reported from community-based settings within the maternity services [89,103].

### Methodological quality

The methodological quality of the 22 included studies was considered good using the JBI checklist for qualitative and quantitative studies [83] and the MMAT tool for mixed-methods studies [84] (see Supplementary file 2). The studies were of good quality, achieving over 88% of the JBI quality criteria. The mixed-methods studies (n = 2) met all five MMAT criteria and were rated as good quality. None of the studies were excluded based on quality ratings but this was taken into consideration during the analysis and synthesis when deciding how much weight to put on the findings of each study which underlines the strength and credibility of our findings to a certain extent.

### Thematic analysis

**Fig 2:**
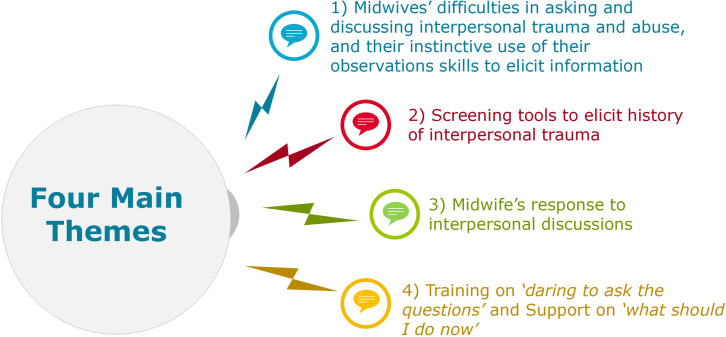
Main Themes.

Data Analysis produced four main themes: Theme 1, Midwives’ difficulties in asking and discussing interpersonal trauma and abuse, and their instinctive use of their observation skills to elicit information. Theme 2, Screening tools to elicit history of interpersonal trauma. Theme 3, Midwife’s response to interpersonal discussions. Theme 4, Training on ‘*daring to ask the questions’* and Support on *‘what should I do now’*. To illustrate synthesis, the main themes are narratively presented with the addition of a thematic summary diagram (Fig 2) and detailed information on the analytic themes and supporting quotations is provided in Table 2.

**Table 2:** Analytic Themes and Supporting Quotations.

Studies:	Examples of assessment questions on interpersonal trauma:	Screening Approach and Measurement tools used:	Midwife’s response to assessing for interpersonal trauma
Mezey et al., (2003) [101]	None reported	Screening was conducted three times: at booking, at 34 weeks and once during the postpartum (within 10 days) using a variation of the Abuse Assessment Screen.	Midwives in this study reported on the benefits of engaging with this topic but reported on many barriers to effective screening such as lack of training, time constraints and the emotionally sensitive nature of the topic. Some midwives felt that screening for domestic violence meant that they were being pushed into yet another new role which they felt they were poorly equipped. Midwives often expressed fears of being assaulted by partners (Mezey et al., 2003). *“Because I’ve been in two violent relationships already, so asking the questions sort of brought it all back for me. Although it’s in the past and I’ve put it to the back of my mind, it’s difficult to ask the woman and try and help them without it creeping back again, and then it’s hard to deal with. It’s not just me, I know a lot of colleagues who’ve been in violent relationships and find this study difficult. But there does need to be support for midwives too if you’re going to try and implement this sort of thing” (*Mezey et al., 2003 p.748-749)
Mortimore et al., (2021) [93]	None reported.	Midwives introduced the ACEs screening tool to booking women and their partners. M card; a small folded leaflet was given to women with the booking pack to explain the meaning of adverse childhood experiences (ACEs) and the five ways to wellbeing, a self-help aid to reduce the impact of ACEs. As well as being an aid to parents, this tool was used by midwives to help start the conversation about ACE.	Most midwives felt comfortable asking the ACEs questions at booking. Self-reports from midwives indicated that, for some, this new knowledge had a positive impact on both their personal life and professional practice. However, if a midwife had experienced ACEs themselves, it was difficult for them to have a conversation with the parents. The rates of disclosure for one or more ACE were 57%, higher than that described in the general population.
McKenzie-McHarg et al., (2014) [86]	None reported	Pink sticker system– to identify women who may be experiencing mental health and/or psychological issues. No specific screening tools were used to assess for trauma even though vulnerable women were identified.	There was some feeling among the midwives that by assessing the need for psychological support and help antenatally, there may be a decreased risk of adverse postnatal outcomes.
Stenson et al., 2005 [94]	Have you ever been emotionally or physically abused by your partner or someone else important to you? Are you living with someone who has threatened you or hurt you physically? Since you have been pregnant, have you been hit, kicked or shoved or otherwise physically hurt by someone? Do you feel controlled or isolated by your partner? Have you ever been forced to participate in or be subjected to sexual acts against your will?	Psychosocial Assessment- also asked trauma questions as part of the psychosocial assessment. The questions were derived from the Abuse Assessment Screen (Soeken et al., 1998)	The midwives described how their role in caring for abused women is to listen to their stories, give emotional support, inform about resources or arrange contacts for additional help and observe subsequent developments. Feelings of failure and frustration were expressed over the fact that not all women were assessed.
Rollans et al., 2013 [88]	10. Now that you are having a child of your own, you may think more about your own childhood and what it was like. As a child were you hurt or abused in any way (physically, emotionally, sexually)?11. Within the last year have you been hit, slapped, or hurt in other ways by your partner or ex-partner? 12. Are you frightened of your partner or ex-partner? Are you safe here at home?/to go home when you leave here? 14. Has your child/children been hurt or witnessed violence? 15. Who is/are your children with now? 16. Are they safe? 17. Are you worried about your child/ children’s safety?	Antenatal psychosocial assessment & depression screening	Modification of questions may reflect a level of discomfort on the part of the midwife in asking sensitive questions and may impact the integrity of the assessment
Hindin, 2006 [103]	None reported	None of the participants used a standardized screening assessment tool, such as the Abuse Assessment Screen, but they adapted their questions based on content in this tool and their individual clinical style. However, they all depend on clinical suspicions or that “gut reaction” throughout pregnancy, during labor, and in the postpartum period in place of routine screening to alert them to potential abusive situations.	Despite their frustrations and lack of knowledge about the outcomes for women, the midwives hold on to the idea of making a difference in the lives of the women they touch. When talking about her practice one midwife said, “If I can make a difference...even for one day, that she feels valued and important and loved...I am doing what I need to do.”
Lazenbatt et al. (2009) [96]	None reported. Midwives (n = 488) in the study by were asked how many had actually raised the issue of domestic violence with women, only 38% of the cohort reported that they raised this issue. The biggest hindrance recorded in the free-text responses for both hospital (78%) and community (89%) was the reluctance of a partner to leave the consultation.	None reported.	Midwives (n 448) based in both hospital (92%) or community (93%) setting felt they had a role to play in responding to domestic violence
Lazenbatt and Thompson-Cree, (2009) [100]	This study reported that 28% of midwives who asked specific questions on domestic violence, 46% (n = 62) reported to have asked a direct question, whilst 8% (n = 11) asked both a direct and indirect question. There were no examples provided on questions asked by midwives.	No formal screening tool used. Only 13% of the sample actually asked a woman a direct question about DV.	Not reported
Eustace et al., (2016) [98]	Some midwives asked women directly about domestic abuse at the booking interview. No examples of questions given.	Other midwives used the first booking appointment to administer a self-report screening tool but no reference to what tool was utilised to assess for domestic abuse.	Participants described feeling unsupported in their role when screening women for IPV. “*We all know the manoeuvres to assist a woman with a shoulder dystocia situation so we should have the same sort of step by step things to do, almost tick box things that you can do if someone discloses domestic violence. There should be no question in your mind what the next step should be*.” (Lynda) (Eustace et al., 2016 p.11). *All participants identified that the role of the midwife should incorporate routine screening for IPV. “… “often times seeing a midwife is a woman’s first contact with some regular healthcare and social care as well. So I really think that it should be one of those things that midwives really should be championing and being a leader in”.* (Lyn) (Eustace et al., 2016 p.9).
Mauri et al., 2015 [97]	Not reported	Not reported	Some midwives saw themselves like bridges: they can identify abused women but have to refer them to dedicated professionals. Screening tools designed to detect violence are available, but the midwives had different feelings and conflicting opinions about them. A screening tool might be useful for midwives to remind them of the problem and offer the women an opportunity for disclosure. Others were afraid that women might be offended by the screening question.
Shamu et al., 2013 [92]	None reported	None reported	Midwives drew a line between their clinical work for which they trained and what they called social problems, including violence, which they did not think should be in their clinical practice
Carroll et al., 2018 [74]	Reported on skill in questioning or screening for trauma and other mental health conditions. No examples provided on questions used.	None reported A clear majority of midwives 70% in this study did not report using screening tools to assess mental health.	Overall confidence midwives rated below the midpoint of the scale (M = 4.37, SD = 1.84). Self-reported skill was higher in relation to discussing mood (M = 3.16, SD = 1.04) and anxiety (M = 3.10, SD = 0.99) compared to discussing sexual abuse (M = 2.15, SD = 1.02), intimate partner violence (M = 2.20, SD = 1.06), all of which were rated below the midpoint of the scale. A majority of midwives reported never asking any women about sexual abuse/sexual violence (62.2%, n = 258), intimate partner violence (54.1%, n = 225). However, skills was rated higher on providing support to women who were traumatised by their birth experience (M = 3.50, SD = 0.98) and who were emotionally distressed (M = 3.22, SD = 0.99). Midwives in this study tended towards asking women they considered to have mental health risk factors about experiences of mood disorders, anxiety and past trauma rather than asking all women. Selective screening practices based solely on clinical judgement can result in at-risk women being missed.
Finnbogadótti and Dykes 2012 [87]	None reported	No tool used	The midwives’ expressed fear of reporting domestic violence, as well as lack of knowledge concerning how to handle the situation if women did disclose such violence. The midwives could blame themselves for having missed signs during the pregnancy. Midwives were afraid of reporting to the authorities when the man was very aggressive, due to fear of retaliation to themselves or to their families. Fear of being perceived negatively by the pregnant woman was another important consideration.
Jackson et al., 2009 [95]	None reported	None reported	The majority of midwives had little if any education in this area and felt unable to deal effectively with disclosures of sexual abuse.
Baird et al., 2013 [89]	None reported	None reported	In the cohort there was a tendency to report an increase in confidence in asking about domestic violence, following training in 2005.
Di Giacomo et al., 2017 [90]	The study was about exploring attitudes rather than assessing Trauma		Of the respondents, 51 (80 4%) have taken care of women who suffered violence, and 25 (49%) believe they can detect violence. A tendency to ‘blame’ women, although decreasing, persists; it is higher among male nurses and, in general, among male operators.
Nyberg et al., 2010 [104]	None reported	None reported	Not reported
de Vries et al., 2020 [105]	A total of 203 midwives answered the question whether or not they actively reviewed the presence of FoC during prenatal check-ups. In 57.6% (N = 117/203) of the cases midwives always asked and 40.9% (N = 83/203) of the participants only addressed it in specific situations, depending on history and signals or risk factors of FoC. Midwives were asked whether they were used to asking women about their birth experience in the postpartum period; 81.1% (N = 150/185) confirmed to do so directly or within one week after the birth (100%, Ncommunity = 78/78 vs. 66.7%, Nclinical = 68/102, p =<0.001). During the six weeks’ postpartum check-up all midwives confirmed asking women about their birth experiences.	No formal screening tool used by midwives.	Not reported
Fenne Fredriksen et al., 2021 [106]	Direct Questions used but no examples provided. Inconsistencies in approach. Some wondered if it was their phrasing of the question that was the reason for their failure to expose violence. One midwife felt that her questions were not always good enough. It was therefore important to be self-aware and conscious of the way she phrased her questions. The majority reported that they had to approach the issue from different angles.	No Screening tool used	The participants were inexperienced in asking questions and found it embarrassing. Some of them also dreaded asking such questions. One midwife said she felt that she was now faced with something she did not have the capacity to deal with. Many of the participants felt that they did not have enough knowledge about the issue, and that what knowledge they did have was a bit sketchy. The majority had not learned about the issue during their training. One participant described her frustration with the fact that they all attended different courses with different areas of focus. Some felt uncomfortable asking such questions when they had never met the person before.

#### 1) Midwives’ difficulties in asking and discussing interpersonal trauma and abuse, and their instinctive use of their observation skills to elicit information.

This theme focuses on how midwives are having trauma discussions using both direct questioning and using their innate observation skills. Six studies [74,96,98,100,105,106] provided valuable insights as to how midwives ask about trauma (See Table 2). Midwives in Fenne Fredriksen et al. [106] felt that their questions sometimes were not good enough and wondered if their phrasing of the questions led to non-disclosures of violence. No specific examples of direct questions were provided and it was reported that midwives used different approaches in how and when to ask the questions [106]. A quantitative study by Carroll et al. [74] reported on self-reported skill in discussing sexual abuse (Mean = 2.15, Standard Deviation = 1.02) and intimate partner violence (Mean = 2.20, Standard Deviation = 1.06), both were rated below the midpoint of the scale (Mean = 4.48, Standard Deviation = 1.82), demonstrating midwives discomfort with questioning around these issues. The majority of midwives in this study reported never asking women about sexual abuse/sexual violence (62.2%, n = 258), intimate partner violence (54.1%, n = 225) reasons for this not individually addressed.

There were no examples provided of questions asked by the midwives in Lanzenbatt et al. [96]. Only 38% of midwives reported asking the question about domestic violence, mainly because the partner was in the room [96]. Lanzenbatt and Thompson-Cree [100] reported that 28% of Midwives asked questions about domestic violence, some asked direct questions and others asked indirect questions. No specific examples of questions used were provided [100]. Eustace et al. [98] reported that some midwives asked women directly about domestic abuse at the booking interview. No examples of questions were provided. The majority of participants perceived that the lack of clear processes within their health service added to their worries around asking direct questions about domestic violence [98].

Questioning on fear of childbirth and birth experiences were reported in de Vries et al. [105] with 57.6% (n = 117/203) of midwives reported always asking open questions on fear of childbirth in the antenatal period. In the same timeframe 40.9% (n = 83/203) of midwives reported only asking in specific situations where signs and symptoms were picked up by observation. In relation to birth experiences 81.1% (n = 150/185) of midwives asked directly within one week after the birth. At the six-week postpartum check-up all midwives confirmed asking women about their birth experiences but the types of questions asked by midwives was not reported [105].

Across seven studies [87,91,92,95,97,103,105], midwives stated that they relied on observation skills to detect signs and symptoms for abuse or trauma. In the study by Finnbogadóttir and Dykes [87] domestic violence was often picked up by the midwives in the postnatal period due to visible bruises and disclosures from the women about domestic violence in the household. The visible bruises were described as having something “concrete” to start the conversation about domestic violence. Similarly, in Shamu et al. [92], physical violence was reported to be easier to detect in comparison to other forms of violence because it can be seen therefore is more obvious, it is easier to ask when there is visible evidence one midwife stated:

*“Perhaps if there are quite obvious marks from battering such as some bruises”* (Anna, Midwife, Mutenda Clinic) [92].

Comparable findings were reported in Mauri et al. [97] where most of the midwives reported difficulties in recognising violence unless it has ‘striking effects’ and that it is easier to identify violence by seeing physical signs (bruising) for example one midwife reported:

“*Well… I think that physical signs might be easier to recognise… but I’m not so sure because I have never met a battered woman who disclosed to me… so… besides physical signs – such as bruises – I don’t know what else I could notice… “* (interview No. 2: MIDWIFE, p.499) [97].

Midwives instincts were used to recognise psychological signs of abuse such as insecurity, anxiety, fear, low self-esteem, they did not formally screen for these but depended on the midwife’s “gut feeling” [97]. Midwives also reported how the use of observation skills to detect domestic violence was further refined by three triggers [103] that lead to “gut reactions” or suspicions of abuse. These included 1) behavioural clues, 2) physical signs and symptoms and 3) cultural clues for example one midwife stated that:

*“Sometimes, honestly, just a gut reaction and the gut reactions of my co-workers as well, the other midwives I work with. And as we are meeting and we are discussing patients we say...something is not right here so it is that gut…. and if that is all we relied on, we would miss opportunities.... p.218* [103].

A study by Jackson et al. [95] explored midwife’s ability to identify women with a history of sexual abuse and discussed both the physical and the psychological signs of sexual abuse. Most comments were related to women’s reluctance, refusal, fear of or difficulty with procedures such as: ‘*Very, very distressed when needing a vaginal examination’s,* p.257 [95]. Midwives in this study described a range of descriptors on how to recognise the signs and symptoms of survivors of sexual abuse such as: women feeling frightened, distressed, distraught, fearful, afraid, anxious, tense, nervous, agitated and tearful. Jackson et al. [95] also explored the physiological indicators that midwives use to observe for sexual abuse, these included: vaginal discharge, bruising, swelling, recurrent urinary tract infections, and recurrent or early history of sexually transmitted infections, vaginal lacerations, history of dyspareunia, vaginismus, frigidity and history of termination of pregnancy. Midwives emphasized the importance of observation skills when screening and assessing for trauma as they serve as a starting point to determine which women may benefit from follow-up care and a referral for psychological support or treatment [95].

Midwives’ perceptions of fear of childbirth (FOC) among women was a key focus in Salomonsson et al. [91] study. Midwives reported that women were more inclined to report their FOC with or without being asked and symptoms such as panic, crying and the need for continuous personal support during labour demonstrated how midwives were using their observation skills to detect fear of childbirth [91]. A quantitative study by de Vries et al. [105] found that a high number of midwives cared for women with FOC. It was reported that 82.8% (n = 207/250) of the midwives saw > 5 women with FOC in a given a year. They also found that, 77.8% (n = 200/257) of midwives recognised psychological signals in relation to the symptoms of fear of childbirth, whilst 59.5% (n = 153/257) reported being able to recognise the physical signs. The same study also looked at PTSD and midwife’s ability to recognise symptoms. Of the participants 69.8% (n = 157/225) could identify one or more DSM-5 (APA, 2013) criteria whereas 18.2% (n = 41/225) could not name any signs or symptoms of PTSD.

#### 2) Screening tools to elicit history of interpersonal trauma.

This theme outlines the screening tools used in the included papers. Six of the included studies [86,88,93,94,98,101,103] reported on trauma screening tools or validated trauma outcome measures used by midwives (see Table 2). Despite 14 studies reporting on the topic of domestic violence only two studies [94,101] utilised the validated Abuse Assessment Screen (AAS) [107] which detects domestic abuse (intimate partner violence) in pregnant and non-pregnant women. An ACE screening tool was utilised by midwives to detect history of ACEs in pregnant women in Mortimore et al. [93]. Most midwives felt comfortable asking the ACEs questions at booking. However, if a midwife had experienced ACEs themselves, the task was more difficult. This tool was well received by HCPs and women in the study [93]. Two studies [88,94] screened for a history of trauma using psychosocial assessments with some questions adapted from AAS [107]. Similar findings in Hindin et al. [103], no specific screening tool was used but questions were based on the AAS [107] and their own clinical style. Finally, a study by McKenzie-McHarg et al. [86] did not use a specific screening tool to assess for trauma but did use a pink sticker system to identify women with mental health concerns.

#### 3) Midwife’s response to interpersonal trauma discussions.

This theme explores midwives responses to having trauma discussions with women with a history of interpersonal trauma. Lack of confidence in relation to assessment was reported by three qualitative studies [87,94,98]. Midwives in Eustace et al. [98] acknowledged feelings of being unprepared and unsupported in their role, leading some midwives describing genuine feelings of fear and anxiety around the possibility of a positive disclosure of intimate partner violence:

*“We are potentially opening this can of worms here; I’m asking a woman some really difficult questions, what on earth will I do with the answers? If I can’t do anything then I should not be asking, and if I’m not ready to deal with some pretty nasty disclosures then I shouldn’t ask.” (Alison, p.11*) [98].

In relation to dealing with definitive disclosures of sexual abuse (56%/n = 207) midwives, responded either ‘no’ or ‘definitely no’ that they could not deal with a disclosure of sexual abuse [94]. Midwives’ expressed fear of reporting domestic violence, as well as lack of knowledge concerning how to handle the situation if women did disclose such violence [87]. The midwives sometimes blamed themselves for having missed signs of domestic violence during the pregnancy and for not being aware of what the woman had been going through. Midwives were also afraid of reporting to the authorities when the man was very aggressive, some reported fear of being perceived negatively by the woman if they questioned about domestic violence. Midwives recognised that avoidance of questions on the experience of violence during pregnancy may be regarded as a failing not only the pregnant woman but also to the unprotected and unborn baby [87]:

“*There are probably many I have missed, for sure” (Focus group 2,* p.184).

Midwives in Carroll et al. [74] did not feel confident in discussing sexual abuse and intimate partner violence with scores reported as (Mean = 2.15, Standard Deviation = 1.02) for sexual abuse and (Mean = 2.20, Standard Deviation = 1.06) for intimate partner violence, all of which were rated below the midpoint of the scale [74]. Midwives in Rollans et al. [88] often modified their questions demonstrating their level of discomfort with the topic. In relation to screening midwives felt they were forced into a new role and were not equipped for it. They also often feared for their own safety and the safety of their families following disclosures [101,106]. Many midwives in Fenne Fredriksen et al. [106] study reported how they felt embarrassed asking questions about domestic violence. A midwife in the study reported that she felt that she did not have the capacity to deal with such a disclosure of violence [106].

Other studies reported on midwife’s experience when caring for women with trauma histories. Midwives reported positive experiences when caring for women with Fear of childbirth (FoC 83.0%, N = 176/212) and Post Traumatic Stress Disorder (PTSD 74.2%, N = 158/213) [105]. This contrasts with Salomonsson et al. [91], where the midwives (n = 21) reported the challenges in meeting the women with FOC professionally, because it is emotionally demanding. Midwives also reported how FOC can be time consuming and described how sometimes women’s fearful behaviour could provoke anger in the individual midwives. They also described how birth plans may invoke a negative attitude towards the woman among midwives. Meeting women with fixed ideas on her labour can be difficult for midwives as it can be stressful to meet their expectations. Despite this, the midwives pointed out the importance of identifying women with FOC and providing professional support adjusted to women’s individual needs [91].

#### 4) Training on ‘daring to ask the questions’ and support on ‘what should I do now’.

This theme investigates how training and support can facilitate HCP’s in their role in working with women who have experienced trauma. In order to implement organisational change to support midwives in promoting trauma screening and assessing, midwives require specific training, knowledge, organisational support and clinical supervision in trauma care practices [90,93,95,99,101]. Qualitative studies highlighted the need for trauma training and clinical supervision [86–88,92,94,97]. The majority of midwives (n = 347) perceived that increase in education, organisational support and reflective supervision would improve their confidence and quality of care they provided to women with history of trauma.

*“We could all benefit in some really good solid training. If there was more guidance for us, then we would feel more confident about asking these questions.”* (Lyn, p.13) [98].

***Training and Education:*** Jackson et al. [95] found that many midwives had little if any education in the area of disclosing sexual abuse and felt unable to deal effectively with disclosures. Midwives felt that basic skills such as listening were all they could offer as a response to disclosures.

*“It is not an area in which I have had any experience and not an area I feel particularly comfortable with, the only thing I feel I could offer is a listening ear”* p.260 [95].

Midwives interviewed in a qualitative study by Fenne Fredriksen et al. [106] felt their knowledge about domestic violence and screening was “sketchy” [106]. The majority reported not having any training on this issue during their training. There was also a concern raised by one participant about the lack of a standardised training programme. The midwifes felt that courses available in practice had different areas of focus and that midwives were approaching the subject differently as a result of different angles covered on training programmes [106]. Carroll et al. [74] reported on midwives perceived lack of knowledge and skill in relation to PTSD. Those without any perinatal mental health education rated lower on recognising PTSD (Mean = 2.18, Standard Deviation = 1.02) compared to those who did receive training (Mean = 2.73, Standard Deviation = 1.05). This demonstrated that specialised education programs can have positive effects on training midwives to work with women who have been affected by trauma in their pregnancy.

This was also echoed by Baird et al. [89] who provided midwives with specific training programs for dealing with women’s disclosure of domestic violence. Participants in Baird et al. [89] study were asked to reflect on their domestic violence training in 2004/2005 and the effect it had on improving their knowledge of domestic abuse in general. This was collated using the indicators “a great deal; a moderate amount; a minimal amount; not at all, or unsure” to assess improvements in knowledge. Increased confidence in *‘how to deal with a positive disclosure’* of domestic abuse was reported when comparing 2010 data with 2005 data that was pre-training. In 2010, 39.5% of midwives reported *‘a great deal’* compared to 21.1% in 2005 (p¼0.034) [89]. Midwives in 2010 were 2.4 times more likely to report ‘*a great deal’* than those in 2005 (odds ratio ¼2.44, 95% confidence interval ¼1.06–5.63). This demonstrates that improvements in knowledge have been maintained following training [89]. Baird et al. [89] also reported on the effectiveness of training on midwives’ knowledge of screening for domestic violence and found that there was increase in knowledge of screening for domestic violence when comparing the 2010 data with 2005. In 2010, 41.5% of midwives reported being able to routinely enquire about domestic violence at least 80% of the time compared with only 12.7% in 2005 (po0.001) and were also creating more opportunities to ask women about abuse than in 2005 [89]:

“*Yes, I would say that midwives feel much more confident in asking now”* (Midwife 5, p.1007).

***Continuity of Care:*** Fragmented and busy maternity care systems with a lack continuity of care for women are proposed as mitigating factors against midwives screening for trauma, dealing as they are with heavy and complex caseloads [97,98,103]. Many of the midwives (n = 59) highlighted the importance of building a relationship with the woman to identify violence [97,98,102,103,106]. The ability to establish a trusting relationship is a key component for successful intimate partner violence screening and one of their greatest screening strengths [97,98,103,106]. Some midwives demonstrated discomfort asking trauma questions at initial booking, they felt uncomfortable asking these sensitive questions when they had never met the woman before and felt that screening would be more appropriate when they had built up a trusting relationship with the woman [102,106].

*“If you don’t build an intimate and trusting relationship with the woman, she will never disclose abuse. You could ask any question you want but she would never say a word…” (interview 8: senior midwife, p.501)* [97]

*The provision of continuity of care throughout the perinatal period by a known midwife enhances the likelihood of effective routine enquiry across time; and provides opportunities to link women with support services and to bolster their social support network (p.14)* [98].

***Referral Pathways and working with the Multidisciplinary Team:*** Another key aspect for midwives (n = 1426) was the ability to respond effectively to positive disclosure and making appropriate referral to another caregiver which involves working collaboratively with the multidisciplinary team and wide range of agencies [74,89,95,97,99,105]. For instance, midwives reported:

*“Collaborating in such situations is always useful: it helps us and it helps the women” (interview No. 1: midwife* p. 502) [97].

‘*I would probably prefer to refer to someone with training in this field. I would be worried about making an inappropriate response and making the situation worse’ p.260* [95].

Midwives reported having liaison meetings with colleagues as a form of support [106]. Meeting their supervisor and asking advice on how to raise and phrase questions on domestic violence was seen as beneficial to junior colleagues [106]. Baird et al. [89] found that training increased knowledge of the referral process and collaboration with other agencies. This was supported by Carroll et al. [74] relating to consulting with and/or refer to other professionals or services, the mean ratings were above the midpoint to the scale colleagues (Mean = 3.97, Standard Deviation = 0.98), managers (Mean = 3.87, Standard Deviation = 1.06) and perinatal mental health services (Mean = 3.61, Standard Deviation = 1.16), apart from discussing referral to child protection services (Mean = 2.51, Standard Deviation = 1.14) [74]. Shamu et al. [92] found that midwives frequently did not take any action once they became aware of cases of violence other than just noting them as social problems. On other occasions, midwives responded by reporting some cases of violence to a referral (tertiary) hospital or to the police. Midwives mentioned referral to a non-governmental referral centre for women who experience gender-based abuse [92]. The main obstacles to reporting IPV identified by Shamu et al. [92] were lack of education, lack of screening tools, conflicting opinions on whether IPV is a social problem, a healthcare problem or the norm.

## Discussion

The authors examined the role of midwives in trauma discussions with women in the perinatal period as reported by the literature. Synthesis of these findings adds to the body of evidence in this area, incorporating recommendations to enhance practice and care of women who have experienced trauma. It is important to distinguish between screening and assessment to contextualise the findings and make them more meaningful to practice. Screening identifies people at risk of a condition but is not diagnostic whilst assessment is more in depth, can be diagnostic and can aid treatment [44]. All of the twenty-two included studies reported on midwives screening for trauma in the perinatal period, but none reported on midwives assessing for trauma. This may be explained by assessment for trauma being outside the midwives’ scope of practice. Across the studies there is variation on how and when to screen for trauma and there was a lack of a clear process to guide broaching such a sensitive subject within the various health care settings. Midwives in the included studies clearly indicated the importance of asking women specific questions to elicit their experience of trauma [74,96,98,100,105,106]. This finding is corroborated in the literature where women report wanting to be asked about abuse, but many health professionals do not feel comfortable or confident asking about abuse [12,28,74,77].

Of the 22 studies examined, two studies [88,94] provided examples of direct or indirect questions asked to women relating to trauma in the perinatal period and highlighted the sporadic and non-structured screening habits of the midwives interviewed. A more trauma-informed approach to screening questions was provided by LoGiudice et al. [108] such as “*many people I provide care to have a history of sexual violence or trauma”* followed by asking about history using sensitive language and finishing with a power shifting statement. Midwives in this study valued direction in using open questioning techniques when dealing with sensitive topics. This was also supported by Paterno and Draughon [109] highlighting that the use of trauma sensitive and non-judgemental language was key to providing a safe space for women to disclose abuse. Carlin et al. [110] presented an argument against using direct questions as they may result in women disengaging from the process, broad questioning was recommended in its place. Good communication skills, including the use of sensitive and non-judgemental language are essential to the delivery of high-quality healthcare [111,112]. Midwives demonstrated their competence in using their observation skills to detect signs and symptoms of abuse in many of the included studies [87,91,92,95,97,103,105]. The value of the midwife’s observation and communication skills, developed via their experience and knowledge cannot be underestimated when having trauma discussions in the perinatal period. These are skills midwives use in everyday practice and are transferable for use in screening. Listening skills and non-judgemental responses to disclosure are valuable when working with women that have experienced trauma [1,12]. Midwives need to recognise and be empowered to use their core communication skills as an integral tool in managing trauma as this review demonstrates that midwives have many essential skills which are transferable to the effective screening for trauma. A recent review [31] found that many women will not disclose their trauma histories to professionals, unless a therapeutic relationship has been established with the professional. This copper fastens the importance of establishing therapeutic relationship with all women, and the development of therapeutic relationships is a priority for all midwives when providing care.

Despite their communication expertise, lack of confidence in screening for trauma was reported by many of the midwives within the review [87,94,98]. Many midwives felt unprepared, unsupported and afraid when screening for trauma and often blamed themselves for missing signs of violence when abuse was reported. Similar findings were reported by nurses in Poreddi et al. [113,114] reporting lack of confidence in screening for violence and attributing this to inadequate training. In the UK routine enquiry for domestic abuse is part of the national care pathway and it is possible that training has led to changes in some of the findings by Baird et al. [89], but lack of confidence was reported in several of the included studies, which echoes recent research [87,94,98]. Midwives need for trauma specific training and clinical supervision was evident in the findings of this review [86–88,92,94,95,97]. The majority of midwives (n = 347) considered that an increase in education, organisational support and clinical supervision would improve midwife’s confidence, and the quality of care provided to women with history of trauma. This finding is supported by Kirk and Bezzant [75] who note the most dominant barrier to screening by health care professionals is a lack of training and education. Midwives require continuous professional development opportunities that address knowledge, attitudes to perinatal mental health, communication and assessment skills [2,28,115]. Education and training on perinatal mental health among health care professionals has been enhanced in recognition of the morbidity and mortality of perinatal mental health disorders for women and their families [62,76]. However, there is limited education available for midwives and other HCPs to inform and implement impact on trauma-informed care (TIC) [1,116]. Such deficits in education and training of midwives particularly in this area is of concern, as midwives, key providers of care antenatally, intranatally and postnatally, are best placed to provide TIC to women (where appropriate) in the perinatal period [116]. Training improves practice by enhancing competence and confidence and a standardised, evidence-based approach to such education would promote consistency in care provided by midwives [1,106,115].

The potential exposure to secondary trauma or vicarious trauma needs to be acknowledged as an occupational hazard for midwives [117]. Midwives reported their response to a traumatic birth event left them predominantly experiencing horror (74.8%) and feeling guilty (65.3%) about what happened to the woman [117]. More than two-thirds of midwives in this study were present during a birth that involved interpersonal care-related trauma, and of these approximately 17% of midwives met the criteria for PTSD [117]. In recognition of the impact of vicarious trauma, debriefing and support from peers was recommended as benefiting midwives and midwifery students affected by secondary trauma [108]. Shorley and Wong [115] also reported midwives feeling guilty and self-blaming after adverse events and reported lack of support from colleagues and noted organisational responses influenced their ability to deal with the trauma and to stay in the profession. A proposed solution to the effects of vicarious trauma is the use of reflective supervision in practice [28,118], in response to midwives feeling overwhelmed with trauma disclosures. The effectiveness of clinical supervision when carried out in a safe space reported improvements in staff confidence, reduction in stress and burnout, increase in staff retention and satisfaction, and professional sustenance [119,120].

Fourteen of the studies reported on midwife’s experiences of screening women for a history of domestic violence during pregnancy [87,89,90,92,94,96–103,106]. This focus on domestic violence screening is not surprising, considering policy recommendations internationally and nationally and the emphasis on education and training in this area in recent years [26,62]. The remainder of the studies focused on screening for sexual abuse [95], ACE [93], ACE and domestic violence [74,86,88] and traumatic birth/PTSD [91,104,105]. Midwives’ experiences of screening for other types of traumas such as sexual abuse, ACE and birth trauma are poorly represented in the literature in comparison to screening for domestic violence which demonstrates a significant gap in practice impacting women who have or are experiencing trauma other than domestic violence.

Considering that validated screening tools are in existence, only five studies [88,93,94,101,103] reported on trauma screening tools or validated trauma outcome measures used by midwives (see Table 2). Although the Abuse Assessment Screen (AAS) [107] is a well validated tool used to detect domestic abuse in pregnant and non-pregnant women [121–123] only three studies utilised this tool. A study by Mezey et al. [101] used a variation of the tool three times in the perinatal period, Stenson et al. [94] used the trauma questions from the tool as part of a psychosocial assessment and whilst Hindin [103] did not use the tool explicitly but adapted questions from the tool for their study.

Using a valid and reliable tool is important when screening as this has considerable public health benefits relating to the prevention and detection of GBV/IPV [26,69] and ensures evidence best practice is implemented [109]. Other validated screening tools also include the Humiliation, Afraid, Rape, and Kick (HARK) questionnaire suitable for antenatal care settings [124], the ACE screening tool [93], the Antenatal Risk Questionnaire (ANRQ) for perinatal health risk assessment tool for trauma [125], and the short version of the Women Abuse Screening Tool (WAST) for IPV [126]. Whilst these tools (AAS, HARK, ACE, ANRQ, WAST) are recommended, their use by midwives were limited in this review in particular the ANRQ as a screening tool to detect trauma. Despite recommendations for the use of screening in practice there are also some counterarguments presented in the literature. A Cochrane review [127] of screening for IPV found for example that although screening was likely to increase identification it did not increase referrals to support services, reduce IPV, nor had a positive impact on women’s health. The authors concluded that screening for IPV increases identification, but it is a problematic concept when traditional screening criteria are applied, as it is a complex social phenomenon rather than a disease. A scoping review [72] into the evidence base for routine enquiry into ACEs found that HCPs were generally comfortable asking the questions and found it easier than expected. However, this review concluded that enquiry did not usually change the care in the visit or the follow up plan. This Cochrane review [127] focused on women’s experiences of violence found that screening did not reduce IPV post enquiry, and reported on women’s health outcomes post enquiry found no change to the women’s wellbeing when followed up post enquiry.

Screening for ACEs among pregnant women has not been widely implemented in antenatal care often attributed to lack of training and education for HCPs [1,40]. When training and resources on ACE screening was provided to HCPs, a significant improvement in the pre-post pilot scores on the willingness of HCPs to screen and discuss ACE’s with women was demonstrated [115,128]. However, a recent study found insufficient evidence for the implementation of an ACE screening programme [129]. Moreover the same study found limited evidence that the routine enquiry/screening for ACE’s resulted in reductions in morbidity and mortality rates. Further criticisms of the introduction of the ACE screening programme to practice, included advice against placing weight on ACE scores as a means to determine risk or to make treatment decisions for individuals [130]. None of the studies in the review examined the impact on the woman’s wellbeing or service utilization following use of routine enquiry for ACE’s. There is however a potential to cause harm to women by having these trauma discussions in an insensitive manner or when no follow up is provided following disclosure. This demonstrates the need for careful consideration when advocating for the introduction of such programmes.

### Strengths and limitations

To our knowledge, this is the first integrated review which synthesises twenty-two studies on the experiences of midwives screening/assessing for trauma in the perinatal period. This review draws upon the views of a large number of midwives (n = 2615), from a variety of geographical backgrounds, educational levels, years of experience and related to both type I and II trauma in the perinatal period. This review highlights the deficits in screening and assessment of trauma among midwives, impacting considerably on the appropriate care of those women affected by trauma, and gives rise to several recommendations to enhance care in practice. Although women were from various backgrounds and geographic locations, similarities were found in their experiences of screening/assessing for trauma in maternity services, indicating potential transferability of the findings. While this is not the first review to focus on trauma discussions in maternity care [31], it is the first to include quantitative studies. A limitation of the review was the explicit focus on midwife’s experiences of discussing, screening and assessing interpersonal trauma which may have excluded the voice of other HCPs who also have a role in the care of women with history of interpersonal trauma in the perinatal period.

### Recommendations for practice

This review highlighted the need for further education and training for midwives in screening, assessment and how to care for women with a history of trauma in the perinatal period. Mandatory training in relation to screening/having trauma discussions is essential based on the findings and the repeated call for more training and education in this area. The review also identified the sporadic and non-structured screening habits among midwives, calling for a need for a more standardised approach to asking questions about trauma in the perinatal period. Midwives need to be empowered to recognise their contribution to screening for trauma using their existing communication skills, developed via their knowledge and experience of practice, with their valuable contribution to the care women receive commended. Where screening does occur, the limited use of validated screening tools is also an interesting finding and one for consideration for maternity settings. It may be beneficial to consider the use of a standardised screening tool which is effective for trauma, for example, for example AAS, HARK, ACE, ANRQ, WAST. However, further research needs to be carried out on the feasibility of this from a HCPs perspective.

It is important to view trauma discussions as complex and require a whole systems approach, including staff training, continuity of carer, support for staff and evaluation of services [31]. This needs to be sustained to ensure a quality of service and to ensure the 6 key principles of TIC which include safety; trustworthiness and transparency; collaboration and mutuality; peer support; empowerment, voice and choice and cultural sensitivity [14]. A woman who has previously experienced trauma may feel unsafe, and this can be escalated by the care received, e.g., physical examinations or specific pregnancy, childbirth and postpartum trauma [1,12,78]. Therefore, the goal is to find a technique that elicits information that does not re-traumatise women. Further studies are recommended on how trauma is recognised in practice and the effectiveness of screening and assessing for trauma services once introduced. Such research would help further the development of a quality TIC service within maternity services.

### Conclusion

This integrative review has highlighted the variations in having routine trauma discussions within the perinatal care, the need for more streamlined screening and assessing practices, and the urgent need for training and reflective supervision in TIC for all HCPs in their perinatal role. This review adds to the body of knowledge on how midwives are screening and assessing for trauma in the perinatal period. Appropriate trauma discussions should be used so that women can receive the care and appropriate treatment for trauma in order to decrease morbidity and mortality in both mothers and newborns. Despite the awareness of the adverse effects of trauma on women and their families [5,7,13,22,57], the literature [1,26,52] highlights that interpersonal trauma is often not detected by HCP’s in the perinatal care. Midwives as key caregivers to women in the perinatal period are best placed to screen, at a minimum and potentially assess affected women in the perinatal period, highlighting the urgent need for education and training on TIC.

## Supporting information

S1 File Search Terms. (DOCX)

S2 File Critical Appraisal. (DOCX)

S3 File Data Extraction Table. (XLSX)

S4 File PRISMA_2020_checklist. (DOCX)
