# Supplementary material for: An integrative review of how midwives are screening and assessing for trauma in women within perinatal services
Source: PLoS One. 2025 Jul 1;20(7):e0327253. doi: 10.1371/journal.pone.0327253 (PMC12212512; doi:10.1371/journal.pone.0327253)
Supplement: S1 File — (DOCX) [file pone.0327253.s001.docx]

**Supplementary Material File 1:** Search Terms

| **PEO Element** | | **Search terms** |
| --- | --- | --- |
| **Population** | Midwives | Midwifery OR Midwife OR Midwives OR Midwife$ OR Obstetrics nurses OR Midwif* OR Obstetrics nurse |
|  | | **AND** |
| **Exposure** | Women in the perinatal period who have experienced trauma (Type I and II trauma) | MM "Child Abuse" OR DE "Child Neglect" OR MM "Trauma-Informed Care" OR MM "Traumatic Experiences" OR DE "Stress" OR MM "Traumatic Experiences" OR MM "Experiences (Events)" OR MM "Trauma" OR MM "Collective Trauma" OR MM "Posttraumatic Stress" OR MM "Posttraumatic Stress Disorder" OR MM "Trauma Reactions" OR MM "Trauma Screening" OR MM "Trauma Treatment" OR retraumatise OR retraumatize OR retraumatisation OR retraumatization OR re-traumatisation OR re-traumatization OR Neglect* OR Sexual abuse OR sexually abus* OR sexual abus* OR abuse* OR advers* OR Adverse childhood experience OR ACE OR Victim* violence OR maltreat* OR child maltreatment OR mistreat OR emotional abuse OR psychological abuse OR PTSD OR post traumatic stress OR post-traumatic stress OR post- traumatic* OR PTSS OR posttrauma* OR trauma* OR traumatic life event* OR psychological trauma OR stress disorder* OR stress reactions OR Complex PTSD OR Complex Trauma |
|  | | **AND** |
| **Outcome** | Competencies, knowledge and ability to assess for trauma | assessment* OR assess OR referral OR referred OR refer OR knowledg* OR understanding OR comprehension OR competenc* OR skill* OR attitude* |
