# Supplementary material for: An integrative review of how midwives are screening and assessing for trauma in women within perinatal services
Source: PLoS One. 2025 Jul 1;20(7):e0327253. doi: 10.1371/journal.pone.0327253 (PMC12212512; doi:10.1371/journal.pone.0327253)
Supplement: S2 File — (DOCX) [file pone.0327253.s002.docx]

**Supplementary material File 2: Quality Assessment**

| **Joanna Briggs Institute: Qualitative Research Checklist** | McKenzie McHarg et al. [98] | Finnbogaóttir & Dykes [95] | Rollans et al. [100] | Salomonsson et al. [91] | Shamu et al. [92] | Stenson et al. [93] | Mauri et al. [90] | Lauti et al. [97] | Eustace et al. [101] | Hindin [87] | Mezey et al. [102 | Nyberg et al. [99] | Fenne Fredriksen et al. [86] |
| --- | --- | --- | --- | --- | --- | --- | --- | --- | --- | --- | --- | --- | --- |
| Is there congruity between the stated philosophical perspective and the research methodology? | **Yes** | **Yes** | **Yes** | **Yes** | **Yes** | **Yes** | **Yes** | **Yes** | **Yes** | **Yes** | **Yes** | **Yes** | **Yes** |
| Is there congruity between the research methodology and the research question or objectives? | **Yes** | **Yes** | **Yes** | **Yes** | **Yes** | **Yes** | **Yes** | **Yes** | **Yes** | **Yes** | **Yes** | **Yes** | **Yes** |
| Is there congruity between the research methodology and the methods used to collect data? | **Yes** | **Yes** | **Yes** | **Yes** | **Yes** | **Yes** | **Yes** | **Yes** | **Yes** | **Yes** | **Yes** | **Yes** | **Yes** |
| Is there congruity between the research methodology and the representation and analysis of data? | **Not clear** | **Yes** | **Yes** | **Yes** | **Yes** | **Yes** | **Yes** | **Yes** | **Yes** | **Yes** | **Yes** | **Yes** | **Yes** |
| Is there congruity between the research methodology and the interpretation of results? | **Yes** | **Yes** | **Yes** | **Yes** | **Yes** | **Yes** | **Yes** | **Yes** | **Yes** | **Yes** | **Yes** | **Yes** | **Yes** |
| Is there a statement locating the researcher culturally or theoretically? | **Yes** | **Yes** | **Yes** | **Yes** | **Yes** | **Yes** | **No** | **Yes** | **No** | **No** | **Yes** | **No** | **Yes** |
| Is the influence of the researcher on the research, and vice- versa, addressed? | **Yes** | **Yes** | **Yes** | **Yes** | **Not clear** | **Yes** | **No** | **No** | **No** | **No** | **Not clear** | **No** | **No** |
| Are participants, and their voices, adequately represented? | **Yes** | **Yes** | **Yes** | **Yes** | **Yes** | **Yes** | **Yes** | **Yes** | **Yes** | **Yes** | **Yes** | **Yes** | **Yes** |
| Is the research ethical according to current criteria or, for recent studies, and is there evidence of ethical approval by an appropriate body? | **Not clear** | **Yes** | **Yes** | **Not clear** | **Yes** | **Yes** | **Yes** | **Yes** | **Yes** | **Yes** | **Yes** | **Yes** | **Yes** |
| Do the conclusions drawn in the research report flow from the analysis, or interpretation, of the data? | **Yes** | **Yes** | **Yes** | **Yes** | **Yes** | **Yes** | **Yes** | **Yes** | **Yes** | **Yes** | **Yes** | **Yes** | **Yes** |

| **Joanna Briggs Institute: Cross-Sectional Studies Checklist** | **Di Giacomo et al. [85]** | **Carroll et al. [69]** | **de Vries et al. [84]** | **Lazenbatt et al. [89]** | **Ali et al. [83]** | **Lazenbatt and Thompson-Cree [88]** | **Jackson et al. [96]** |
| --- | --- | --- | --- | --- | --- | --- | --- |
| 1. Were the criteria for inclusion in the sample clearly defined? | **Yes** | **Yes** | **Yes** | **Yes** | **Yes** | **Yes** | **Yes** |
| 1. Were the study subjects and the setting described in detail? | **Yes** | **Yes** | **Yes** | **Yes** | **Yes** | **Yes** | **Yes** |
| 1. Was the exposure measured in a valid and reliable way? | **Yes** | **Yes** | **Yes** | **Yes** | **Yes** | **Yes** | **Yes** |
| 1. Were objective, standard criteria used for measurement of the condition? | **Yes** | **Yes** | **Yes** | **Yes** | **Yes** | **Yes** | **Yes** |
| 1. Were confounding factors identified? | **No** | **No** | **Yes** | **Yes** | **No** | **Yes** | **Yes** |
| 1. Were strategies to deal with confounding factors stated? | **No** | **No** | **No** | **Yes** | **No** | **Yes** | **Yes** |
| 1. Were the outcomes measured in a valid and reliable way? | **Yes** | **Yes** | **Yes** | **Yes** | **Yes** | **Yes** | **Yes** |
| 1. Was appropriate statistical analysis used? | **Yes** | **Yes** | **Yes** | **Yes** | **Yes** | **Yes** | **Yes** |

| **Mixed-Method Studies using the Mixed Methods Appraisal Tool** | **Baird et al. [94]** | **Mortimore et al. [103]** |
| --- | --- | --- |
| S1. Are there clear research questions? | **Yes** | **Yes** |
| S2. Do the collected data allow to address the research questions? | **Yes** | **Yes** |
| Is there an adequate rationale for using a mixed methods design to address the research question? | **Yes** | **Yes** |
| Are the different components of the study effectively integrated to answer the research question? | **Yes** | **Yes** |
| Are the outputs of the integration of qualitative and quantitative components adequately interpreted? | **Yes** | **Yes** |
| Are divergences and inconsistencies between quantitative and qualitative results adequately addressed? | **Yes** | **Yes** |
| Do the different components of the study adhere to the quality criteria of each tradition of the methods involved? | **Yes** | **Yes** |
